# Role of a Community-to-Community Learning Strategy in the Institutionalization of Community Mobilization among Female Sex Workers in India

**DOI:** 10.1371/journal.pone.0090592

**Published:** 2014-03-07

**Authors:** Santhosh Sadhu, Archana Rao Manukonda, Anthony Reddy Yeruva, Sangram Kishor Patel, Niranjan Saggurti

**Affiliations:** 1 CARE India, Hyderabad, India; 2 HIV and AIDS Program, Population Council, New Delhi, India; University of Illinois at Chicago, United States of America

## Abstract

**Introduction:**

The institutionalization of community mobilization is not well understood in literature. This paper aims to understand the role of the community-to-community learning strategy in the institutionalization of community mobilization among sex workers communities across eight districts of Andhra Pradesh, India.

**Materials and Methods:**

Data collected during baseline (March, 2010) and endline (June, 2012) under an HIV prevention project (SAKSHAM project) was used to investigate the strength (as score) of community mobilization based on two learning strategies: non-government organization (NGO)-to-community-based organization (CBO) strategy, and community-to-CBO strategy. The strength of community mobilization was assessed based on different parameters. The change in scores were computed as a percentage of the improvement to the total potential improvement from baseline to endline on specific indicators and overall.

**Results:**

Most of the CBOs considered in the pre-post assessment had been registered during 2004–2008. At baseline, the community ownership and preparedness index scores for the eight CBOs under the community-to-CBO strategy ranged between 21.5 and 27.7 while the scores for the three CBOs under the NGO-to-CBO strategy ranged between 16.3 and 21.5. By endline, the strength of community mobilization among CBOs under the community-to-CBO strategy increased 18 points (equivalent to 23% potential improvement) whereas the strength of community mobilization among CBOs under the NGO-to-CBO strategy increased only 10 points (equivalent to 13% potential improvement). The average percentage difference in improvement between the strategies was 10% (p = 0.102). Further analyses indicate that a greater improvement in community-to-CBO learning strategy was noted around managerial capacities and engagement with stakeholders than other parameters.

**Conclusion:**

The community –to- CBO learning strategy presents promising results for HIV prevention with regard to institutionalization of community mobilization among sex workers communities. Findings support the scaling-up of community mobilization initiatives within HIV prevention interventions using well trained community members in India and elsewhere.

## Introduction

Over the years, community mobilization has become an integral part of health and development interventions [1], [2], [3], particularly in the context of the growing need for sustainable HIV prevention interventions and shrinking resources in concentrated HIV epidemic settings such as India. One of the strategies for the promotion of community mobilization [4], [5], [6] in HIV prevention interventions in India has been through the use of non-governmental organizations (NGOs). These organizations have been established under the society act or legal profit companies, as per the Indian government guidelines [6]. In India, community mobilization efforts by NGOs have been in the nature of setting up community-based groups or organizations (CBOs) where individuals from the target population (such as female sex workers) come together to lead and implement HIV prevention interventions [7], [8], [9], [10]. However, such community mobilization efforts have not been able to adequately cover the huge volume of key populations groups at HIV risk: female sex workers (FSWs) (approx. 0.9 million) and men who have sex with men (MSM) (approx. 0.4 million) in India. More importantly, the traditional practice of NGO-led interventions, where professional outreach staff guiding community responses, have been proven to be inadequate for the development of a deeper understanding of the context in which communities live and operate [4], [5], [9].

In this context, a unique effort of engaging community members to mentor communities (also referred to as community-to-CBO learning strategy) was undertaken during 2010 for the first time in southern India. This community-to-CBO learning strategy builds on research which demonstrates that community engagement has the potential for rigorous program planning, decision-making and participatory evaluation [6], [7], [11], [12]. More specifically, these studies have shown that communities informally or formally encourage other community members not only to participate in intervention strategies, but also to become self-sufficient in program planning and implementation [7], [12], [13].

Our search for literature neither finds studies describing the mentor role of the community members in strengthening CBOs nor the comparison of the effect of community mentoring with traditional NGO-led mentoring for CBO strengthening. Keeping this in view, our paper aims to assess the effect of the community-to-CBO learning strategy and address the research gap by exploring: (a) the role of the community members in strengthening of CBOs; and (b) the relative advantages of the NGO-led learning strategy and the community-led learning strategy in strengthening of CBOs and/or community mobilization.

### Program Design

The community-to-CBO learning strategy under the SAKSHAM program incorporates community engagement to strengthen the leadership and managerial capacities of community based organizations. The design of the community-to-CBO learning strategy was guided by the social learning and diffusion of innovation theory, in which the process of behavioral practices and skills are fostered by social interaction [14], [15], [16]. The application of this theory is adapted to organizational diffusion, which is a process by which change in the strength of organizations occurs through the communication of ideas or the demonstration of new methods [14]. The goal of using this peer-based mentoring is to help target population gain the confidence to fight for their rights and learn the skills to manage the program on their own.

The community-to-CBO learning process comprised seven steps, discussed below. The process aimed to build the community's capacity to understand the national AIDS control organization (NACO) program guidelines on community mobilization interventions, and to develop their leadership skills, managerial capacities, and engagement with other stakeholders, so as to improve the strength of community mobilization.

1. **Identifying learning sites.** The first step in the process was to identify learning sites, which are defined as intervention sites that are successful in terms of community mobilization; that is, they have formed CBOs where community are engaged in the target intervention (TI) program management, and have demonstrated an improvement in core indicators related to HIV prevention. To identify learning sites, a working committee was formed consisting of state-level program managers; the committee worked together with community members and researchers to assess the performance of the CBOs on the following five parameters, developed as part of the learning sites assessment tool [17]: (1) leadership, problem solving and decision-making; (2) systems for community self-management of interventions; (3) community participation and ownership leading to increased performance in TI core indicators; (4) documentation strength; and (5) representation, recognition and awards. Through these parameters, the tool measures the fundamental principles of participation, inclusion and sustainability with regard to community mobilization. Details of the learning site assessment tool are given in Table 1, and the principles of preparing this tool has followed the previously published work on measurement of strength of community mobilization interventions [3]. Multiple research techniques were used to implement the tool, including interviews with program personnel (CBO representatives, office bearers and outreach workers), group discussions (with peer educators), and the validation of information through secondary documents, fact sheets, and interviews with community members from the learning site. The information generated from this tool was then scored on a 0–3 point scale qualitatively by the working committee administering the tool based on validation with evidence and team consensus. The parameter scores were then calculated, and all the assessed sites were categorized under three band definitions (as per discussions of the working committee) based on the score percentage: ‘needs more inputs to be eligible as a learning site (0–50%)’, ‘needs nurturing to develop as a learning site (51–75%)’, and ‘has the capacity to serve as a learning site (76–100%)’. Based on this approach, 10 potential learning sites (CBOs) were assessed, and six identified as suitable sites. Details and results of this approach have been published elsewhere [18].
2. **Selection and capacity building of community faculty.** After the learning sites were identified, six community members, known as the community faculty, were selected, based on their ability to build skills, and their commitment to sharing the learnings with other community members. The selection of community faculty members was also based on inputs from CBOs with learning sites and the institutions guiding those CBOs. Community faculty members were trained to work in partnership with other CBO members, plan interventions, develop community engagement enhancement plans (CEEPs) and design tools for monitoring and evaluating the strength of community mobilization, power dynamics among community members, adult learning techniques and participatory learning techniques. CEEPs are plans for the institutionalization of community mobilization through the active engagement of the community. A ‘learning circuit’ curriculum, which is the curriculum demonstrated by the learning sites, was simultaneously developed.
3. **Organizing on-site capacity building workshops to demonstrate community mobilization learning elements.** On-site capacity building, which is the demonstration by learning sites to visiting site members, was implemented. On-site capacity building workshop participants included community representatives and project team members from beneficiary sites. Sessions were conducted over a period of 3–4 days, and covered various aspects of community mobilization such as understanding the community, self-esteem, attitudinal adjustment, and orientation on the National AIDS Control Program's (NACO) community mobilization strategy. During the onsite capacity building workshops, participants developed CEEPs with the help of the community faculty at the learning sites. Workshops also covered various aspects of power dynamics among community members, and participatory learning tools.
4. **Organizing skills-building workshops for community faculty to enhance their training skills.** During skills-building workshops the community faculties were trained to take the lead in implementing community members' CEEPs in the field. The skills building workshops for community faculty was held for 20 days. During the workshop, the community faculty members were encouraged to share their knowledge and experiences, think critically and solve problems, articulate their ideas, use participatory learning tools, build community skills, and openly transfer learnings to the community members. They were also encouraged to visit well-known community-led organizations implementing HIV intervention projects, such as the Durbar Mahila Samnwaya Committee (DMSC) in Sonagachi and Ashodaya Samithi at Mysore [7], [12], [19], to gain an orientation on the various methodologies for implementing community mobilization programs.
5. **Recruiting a mentoring team and providing on-site mentoring.** While community members' visits to the learning sites were useful, our project additionally provided on-site mentoring. For this purpose, two mentoring teams were set up with six community faculty members from each of the learning sites, with each team comprising one mentor (a professional expert, hired as a consultant) and three community faculty members. The objective of setting up mentoring teams was to enable community members from the CBOs to implement the CEEPs. To build capacity, every month the mentoring team would spend two days with each organization to provide handholding support to roll-out the CEEP, based on day-wise action plans. During this process, the mentoring team interacted with the CBO leadership team to ensure community participation to establish and strengthen community mobilization. Each mentoring team worked with three CBOs on an average in the project.
6. **Reviewing progress through participatory sharing meetings.** In addition to on-site mentoring, participatory sharing meetings were organized with CBOs on a quarterly basis to help them draw up action plans for the quarter and to provide updates on the preceding quarter. Two representatives from each of the participating CBOs, mentoring teams and project technical officers attended the meetings and reviewed the status, achievements and challenges with regard to community mobilization. These meetings have built the confidence of community members to stand up in front of a crowd, and share their experiences, which has resulted in increased self-esteem and a sense of group solidarity. These meetings also helped CBOs work towards implementing CEEPs, as they were expected to share updates during each meeting.
7. **Evaluating the impact of the community development strategy.** The final step of the community-to-CBO learning strategy involved both process documentation and outcome evaluation.

**Table 1:** Summary of learning assessment tool -- used for identifying learning sites.

Parameter 1: Leadership, problem solving and decision making process.
***Definition*** *:* This parameter assesses whether local intervention is led by community, it is independent, with strong systems for democratic, inclusive leadership, and internal governance systems along with participatory decision making mechanism to addresses the basic fundamental need for mobilization of target population, increasing their engagement and ownership in problem solving and decisions making process.
***Indicators: (17 indicators based on 60 questions)***
1.1: CBO has established independent office/Drop In Centre-Clinic and managing on its own.
1.2: The intervention has strong systems for democratic/participatory process, including norms for selection/election of office bearers.
1.3 & 1.4: Process followed during pre-selection stage and during the election stage.
1.5: CBO has systems to ensure governance, leadership rotation and has strong systems for leadership accountability.
1.6: CBO meets regularly to execute its functions.
1.7: Knowledge, information and awareness levels of the CBO office bearers about CBO.
1.8: Office bearers' ability to ensure/writing and dissemination of annual, periodic progress, financial reports and meeting minutes to members.
1.9: Knowledge and information levels of the office bearers on legal and health rights of the HRGs and PLHAs.
1.10: Motivation of office bearers to work for CBO.
1.11: Office bearers' ability to mobilize Resource - internal and external resources.
1.12: Office bearer's role in organizing sensitization programs.
1.13: Office bearer's role in planning and initiating action on crisis, issues and responding to the emergency issues. : Proactiveness of the office bearers in planning and initiating action on crisis, issues and community needs.
1.14–1.17: Decision making style in CBO management.

Parameter 2: Systems for community self-management of the intervention.
***Definition:*** This parameter basically explores whether the systems are functioning to ensure the Community engagement and participation in various activities of service delivery-not just as a beneficiary, but also by having acquired greater control over decisions made in the project spaces such as managing through sub-committees. Community members are also able to create systems which can detect and mitigate risks-legal and financial-in the management of the organization itself, without requiring much support from the NGO.
***Indicators: (5 indicators based on 21 questions)***
2.1: Project monitoring and implementation of activities.
2.2: Formation and functioning of various committees.
2.3: Grievance redressal mechanism and confidentiality policy/and mechanism for conflict resolution.
2.4: Community mobilization and collectivization of HRGs as part of CBO.
2.5: Level of maturity of the CBO members and office bearers in articulating their activities and experiences.

Parameter 3: Community participation and ownership leading to increased performance in TI core indicators.
***Definition*** *:* This parameter elaborates the performance of the particular site with regard to the program core indicators in community participation and ownership perspective. It helps to identify the potentiality of the proposed Learning site to serve as a model site to showcase effectiveness of community mobilization leading to ownership and increased performance in the TI. It envisages to identify demonstrable capacity of community to monitor and strategize service delivery for HIV risk reduction; creating enabling environment to address the issues and needs of the community members, and make progress towards expanding outreach and coverage, promoting positive living and linkages to engage with local and state bodies, solidarity groups to address members' vulnerabilities; and has the potential to influence other stakeholders such as relevant government departments, neighbourhood communities, and the general population.
***Indicators: (6 indicators based on 34 questions)***
3.1: Community ownership of the TI program.
3.2: It determines the effectiveness and significant impact of community mobilization and peer education in performance of TI core indicators, and how community mobilization and empowerment has increased the health seeking behaviour among HRGs?
3.3: CBO initiative in promoting positive living and positive prevention.
3.4: CBO capacity in reducing the vulnerabilities of CBO members negotiating, claiming of rights and entitlements, including sensitization on violence, harassment, and discrimination.
3.5: Community efforts and role in reactive and proactive advocacy and sensitization of stakeholders.
3.6: CBOs assertion with various non-stakeholders and strategic relationships, creating enabling environment for addressing other issues of the community.

Parameter 4: Documentation strength.
***Definition*** *:* This parameter assesses whether the particular site has demonstrable capacity of the document strength to provide evidences related to all the above basic parameters.
***Indicators: (3 indicators based on 29 questions)***
4.1: Organization registration related documents: the team has to check the documents which are not verified.
4.2: Financial management.
4.3: CBO Networking and strategic partnerships with relevant local/district level and national level organizations networks and collectives.

Parameter 5: Representation, recognition and awards.
***Definition:*** This indicator attempts to identify the prominence of the particular CBO, community and any extraordinary achievements for categorising it to serve as a learning site.
***Indicators*** *: (1 indicator based on 5 questions)*
5.1: The basic objective of this indicator is to extract the relevant information of the CBO related to appreciation and recognitions received for its performance, contribution and participation in HIV/AIDS prevention program, which would be useful as an evidence of their performance.

## Materials and Methods

A pre-test and post-test evaluation design was used to investigate the strength of community mobilization based on the two strategies of learning. Specifically, 12 CBOs were enrolled into the evaluation study from five coastal districts of Andhra Pradesh, which were part of either: (a) the community-to-CBO learning strategy (8 CBOs); or (b) the NGO-to-CBO learning strategy (3 CBOs). For the selection of CBOs, a list of TIs in Andhra Pradesh was prepared that were funded by the Andhra Pradesh State AIDS Control Society or a state level NGO to implement HIV prevention programs among FSWs or MSM. The TIs were then categorized as either CBO-led or NGO-led based on who is taking lead in implementing TIs. Due to time and resource constraints, the evaluation has considered to select eight CBOs under intervention group and four CBOs under control group, from among the list of NGO-led TIs. Accordingly, the 12 CBOs (out of 20 CBOs in study districts) were randomly assigned to either receive community-to-CBO learning strategy (intervention sample) or to receive NGO-to-CBO learning strategy (control sample). One CBO from the control sample was excluded as the program was non-functional for over six months during the evaluation study. Interviews were conducted with individuals from the CBO leadership and management team, and key informants from the target population to assess the strength of the CBOs before and after the implementation of intervention.

The evaluation used the community ownership and preparedness index (COPI) tool, which was developed as a cross-sectional tool to assess the strength of community mobilization and the transition readiness of the program to the CBOs from NGOs. The COPI tool was developed by researchers from Praxis Institute for Participatory Practices [3], and have confirmed both construct validity and reliability with its use in various settings. According to Praxis researchers [3]: *The indicators included in the COPI tool measured in the COPI tool categorized into four dimensions at the broadest level: (1) leadership, governance and decision-making; (2) sustainability through resource mobilization and networking; (3) project management; and (4) engagement with the state and wider society.”*

Details of the process of tool development, interview tools, calculation of index scores and interpretation of the series of bands are discussed elsewhere [3]. This tool was adapted for use in the SAKSHAM project evaluation and the field work was done by the Praxis researchers who have developed the COPI tool. The calculation of scores in this study followed similar principles as published in earlier works [3], [6]. However, the overall index scores of the data were computed to determine the strength of community mobilization, and the scores were grouped into a series of four “bands” for the purpose of this study: Needs Improvement (0–30), Generally Meets Expectations (31–55), Fully Meets Expectations (56–80), and Exceeds Expectations (81–100). The difference between the COPI tool used for evaluation and learning assessment tool used for identification of learning sites is that the latter assessed with a view to present the CBO as learning site and their members as community faculty; whereas the former COPI tool measures the actual strength of CBOs. Although, there is some similarity in indicators measured within learning assessment tool and COPI tool (mainly with regard to leadership and managerial skills assessment), large differences exist in the question formats of both the types of tools.

### Ethics Statement

CARE-India, under the guidance of the Andhra Pradesh State AIDS Control Society, provided general oversight and approval for the collection and use of data at the organizational level to assess the value of the community-to-CBO learning strategy with regard to scaling -up community mobilization. Data were obtained from key informants and interviews were conducted in group settings [20]. No personal identifiers were recorded. Participants were informed about the purpose of the study and their right to withdraw at any time during the interview. Verbal consent was taken from each respondent prior to their participation in the discussion. The study involved key informants -- information was taken from these informants but not about them -- and questions were targeted at exploring their opinions and performance at the organizational level; therefore, it was not deemed necessary to get ethical approval for the study from the institutional review board, as the study does not qualify as human subjects research as defined by DHHS regulations 45 CFR 46.102.

### Measures

In this study the COPI tool was used to measure the effectiveness of the community-to-CBO learning strategy as compared to the NGO-to-CBO learning strategy. The COPI tool uses four overarching dimensions of a CBO's capacity (organizational strength; sustainability; program management; and engagement on issues of rights, entitlements and stigma reduction), which are considered essential for transition readiness to sustain HIV prevention strategies. Following a discussion with community members and experts, these four dimensions were subdivided into eight components or ‘parameters’ characteristic of a strong participatory CBO (e.g. (1) leadership, governance and decision-making; (2) sustainability through resource mobilization and networking; (3) program management; and (4) engagement with the state and wider society to reduce stigma), addressing both its internal functioning and external elements that affect its organizational development [20]. These dimensions were divided into six parameters which were further subdivided into 23 specific indicators. Progress on each indicator is assessed through set of questions. Each possible response to a question is pre-coded with a score within the COPI tool. The scores received for the questions relating to an indicator are totalled to arrive at an overall score for that indicator and for the set of parameters.

### Statistical Analyses

The data for the COPI index scores were calculated using the analytical tool published elsewhere [3]. For example, rather than assigning the same score to each CBO where a particular activity takes place (captured using a specific question in COPI tool), a progressively higher score was assigned if the planning for that activity was done by paid peer educators, or by the leadership team working with or without peer educators. Using this procedure, scores were assigned to each aspect of the questions for analyses. For the purposes of this evaluation, the overall COPI scores and the scores for each of the parameters and indicators were calculated as a percentage of the actual improvement to the potential level of improvement (between baseline and endline), and the values compared. These values were presented separately for the two groups: community-to-CBO learning strategy, and NGO-to-CBO learning strategy. Analyses assessed the relative increase on various parameters of strength of community mobilization for the community-to-CBO strategy in comparison with the NGO-to-CBO strategy. Given the small sample size, the difference between the strategies was assessed using the non-parametric test (Mann-Whitney Test) and the significance was set at p<0.15.

## Results

Most of the CBOs considered in the pre-post assessment were registered during 2004–2008. The eight CBOs in the community-to-CBO learning strategy were a little older in terms of the year of registration as compared to those under the NGO-to-CBO learning strategy. Most of the CBOs had an average estimated target population size of 1000, and were primarily implementing the program for the FSW population. At baseline, the COPI scores for the eight CBOs under the community-to-CBO strategy ranged between 21.5 and 27.7 while the scores for the three CBOs under the NGO-to-CBO strategy ranged between 16.3 and 21.5 (Table 2). As per the categorization of COPI [3], all the CBOs were in the foundation stage. By endline, there was an average increase in the COPI scores within both the groups. The strength of community mobilization among CBOs under the community-to-CBO strategy increased 18 points (equivalent to 23% potential improvement) whereas the strength of community mobilization among CBOs under the NGO-to-CBO strategy increased only 10 points (equivalent to 13% potential improvement). The average percentage difference in improvement between the strategies was 10% (p = 0.102).

**Table 2:** Profile of community-based organizations, and their improvement in strength (as score) between baseline (2010) and endline (2012), by type of strategy, Andhra Pradesh.

						CBO score				
Type of strategy/Serial Number of CBOs	District	Name of CBO	Year of CBO Registration	Estimated population size	Typology	BL (2010)	EL (2012)	^#^ Difference	ˆ Change	^$^ Improve-ment
**Community-to-CBO Learning**						**24.6**	**42.2**	**17.6**	**23.3**	**10.4 (p = 0.102)**
1	East Godavari	Udaya Rekha Mahila mandali	2003 (NOV)	979	FSW	27.7	37.7	10.0	13.8	
2	Kurnool	Suraksha Sangam	2006 (AUG)	1100	MSM	21.5	45.6	24.1	30.7	
3	Visakhapatnam	Naandi Service Soceity	2008 (APR)	1491^a^	^*^ Core Composite	23.7	37.2	13.5	17.7	
4	Hyderabad	Udaya Rekha Mahila mandali (URMM)	2005 (FEB)	896	FSW	23.0	36.0	13.0	16.8	
5	Prakasam	Asha Rekha mahila mandali	2006 (FEB)	900	FSW	27.5	48.0	20.6	28.3	
6	Prakasam	Prashanthi mahila sangam	2006 (MAY)	1200	FSW	22.8	45.6	22.8	29.5	
7	West Godavari	Adarsh mahila mandali	2003 (NOV)	800	FSW	26.7	40.0	13.3	18.1	
8	Kurnool	Jeevana Sravanthi Mahila mandali (JSMM)	2004 (DEC)	1250	FSW	23.9	47.4	23.5	30.8	
**NGO-to-CBO Learning**						**19.4**	**29.8**	**10.4**	**12.9**	
9	Cuddapah	Pavitra Mytri Sangam	2007 (OCT)	1500^b^	^*^ Core Composite	16.3	35.5	19.3	22.9	
10	Rangareddy	Asha Charitha Sangam	2008 (MAR)	1200	FSW	21.5	24.4	2.9	3.7	
11	Nizamabad	Raja Rajeshwari Mahila Sangam	2008 (AUG)	1500	FSW	20.4	29.6	9.1	11.4	

Note:^ a^ (FSW-588, MSM-903); ^b^ (FSW-450, MSM-1050); ˆ Change (Actual improvement as percent potential improvement)  =  Actual improvement/Potential for improvement (100 - Baseline score); ^#^ Difference between Baseline-2010 (BL) and Endline-2012 (EL) score; ^$^Percent difference in improvement by strategy; ^*^ Core Composite  =  Combination of FSW and MSM CBOs; p-value were calculated using Mann-Whitney U test for difference between community-to community learning and NGO-to CBO learning.

The study also assessed the improvement in the parameters of CBO strength (Table 3). There was a significant improvement in the managerial capacities of CBOs under the community-to-CBO strategy as compared to those under the NGO-to-CBO strategy (p = 0.025), followed by engagement with other stakeholders (p = 0.102) and overall score (p = 0.102). Although not statistically significant, there was a considerable difference between the two types of groups with respect to the CBO functioning process and status parameter. There was little difference in the scores for other parameters, such as leadership skills, community participation and awareness of the alignment of the program with national guidelines, between the two groups.

**Table 3:** Difference in percent improvement in the parameters between CBOs mentored by communities and NGOs at baseline and endline (2010-2012), Andhra Pradesh.

		Community-to-CBO learning strategy			NGO-to-CBO learning strategy				
Parameter	Maximum Score	BL	EL	ˆ Change	BL	EL	ˆ Change	^$^ Improvement	*p-value
		(2)	(3)	(4)	(5)	(6)	(7)		
Awareness and information levels of community with regard to NACO guidelines on community mobilization strategy	12.0	4.5	5.9	19.3	2.7	4.3	16.9	2.4	0.838
CBO functioning process and status	16.0	5.7	8.5	27.0	6.4	7.5	11.6	15.4	0.221
Managerial capacities	20.0	5.1	10.6	37.3	3.5	4.7	7.2	30.0	0.025
Leadership skills	18.0	3.8	4.5	5.0	2.1	3.8	10.8	−5.9	0.307
Community participation	19.0	3.6	8.3	30.3	3.2	7.1	24.7	5.6	0.540
Engagement with other stakeholders	15.0	2.0	4.4	18.6	1.5	2.4	6.8	11.8	0.102
**Overall score**	**100.0**	**24.6**	**42.2**	**23.3**	**19.4**	**29.8**	**12.9**	**10.4**	**0.102**

*Note:* ***ˆ*** *Change (Actual improvement as percent potential improvement)* ***=*** Actual improvement/Potential for improvement (100 - Baseline score); Baseline survey 2010 (BL) and Endline survey 2012 (EL); ^$^Percent difference in improvement on parameters by strategy; ^*^ p-value were calculated using Mann-Whitney U test for difference between community-to community learning and NGO-to CBO learning.

*Percent improvements were calculated using the following formula:*

Column (4)  =  (Col.(3) - Col.(2))/(Col.(1) - Col.(2)) * 100.

Column (7)  =  (Col.(6) - Col.(5))/(Col.(1) - Col.(5)) * 100.

*Column (8)  =  Col.(7) - Col.(4).*

The data were further analyzed to identify the components of each of the three parameters that improved over time between the strategies for learning. Results, in Table 4, indicate that actual improvement in the CBO functioning process and status as a percentage of potential improvement among CBOs under the community-to-CBO strategy was 27%, whereas the corresponding improvement among CBOs under the NGO-to-CBO strategy was only 11%. Differences were evident, particularly due to the improvement among CBOs under the community-to-CBO strategy, with regard to scores related to the following indicators: operational CBO systems with a greater role to leadership (increased by 37%) and committees formed for TIs and meeting regularly (increased by 9%). Within the NGO-to-CBO strategy, there was a considerable reduction in the score with regard to committees meeting regularly under TIs (declined by 173%). Similarly, under the managerial capacities parameter, there was a significant increase in the community capacity to implement, monitor and strategize project services among CBOs under the community-to-CBO strategy (increased by 41%) as compared to the NGO-to-CBO strategy (which increased by only 9%). The increase in score from baseline to endline is also noted in community mobilization skills among CBOs under the community-to-CBO strategy (increased by 16%), as compared to CBOs under the NGO-to-CBO strategy (decreased by 1%). The results also indicate a considerable improvement in the score of CBOs under the community-to-CBO strategy with respect to engagement with state stakeholders and non-stakeholders. For example, the increase in score with regard to engagement with non-stakeholders among CBOs under the community-to-CBO strategy was 30%, whereas it was only 17% among CBOs under the NGO-to-CBO strategy.

**Table 4:** Differences in percent improvement among the indicators of selected parameters between CBOs mentored by communities and NGOs, at baseline and endline (2010-2012), Andhra Pradesh.

		Community-to-CBO learning strategy			NGO-to-CBO learning strategy			
Parameter and Indicators	Maximum Score	BL	EL	ˆ Change	BL	EL	ˆ Change	^$^ Improvement
	(1)	(2)	(3)	(4)	(5)	(6)	(7)	(8)
**CBO functioning process and status.**	**16.0**	**5.7**	**8.5**	**27.0**	**6.4**	**7.5**	**11.6**	**15.4**
CBO leadership exists and meets regularly to execute its functions	3.0	1.7	2.2	32.1	2.0	2.2	18.8	13.4
Selection process of LT and committee members	6.0	2.0	2.9	23.2	1.9	3.7	44.4	−21.3
CBO systems are operational with greater role to leadership	4.0	0.4	1.7	36.8	0.1	0.4	6.6	30.1
Committees have been formed for TI and committees are meeting regularly	3.0	1.6	1.7	9.2	2.3	1.2	−173.9	183.1
**Managerial capacities**	**20.0**	**5.1**	**10.6**	**37.3**	**3.5**	**4.7**	**7.2**	**30.0**
Capacity of Community committees to implement, monitor and strategize project services	15.0	2.4	7.6	41.1	1.1	2.4	8.9	32.3
Community mobilization skills	5.0	2.7	3.1	16.2	2.3	2.3	−1.2	17.3
**Engage with other stakeholders**	**15.0**	**1.9**	**4.4**	**18.6**	**1.5**	**2.4**	**6.8**	**11.8**
Engagement with state stakeholders	9.0	1.5	2.2	10.0	1.1	1.1	−0.7	10.7
Engagement with non-state stakeholders	6.0	0.5	2.2	30.3	0.3	1.3	17.2	13.2

*Note:* ***ˆ*** *Change (Actual improvement as percent potential improvement)* ***=*** *Actual improvement/Potential for improvement (100 - Baseline score); Baseline survey 2010 (BL) and Endline survey 2012 (EL); ^$^Percent difference in improvement on parameters and indicators by strategies. Percent improvements were calculated using the following formula:*

Column (4)  =  (Col.(3) - Col.(2))/(Col.(1) - Col.(2)) * 100; Column (7)  =  (Col.(6) - Col.(5))/(Col.(1) - Col.(5)) * 100; Column (8)  =  Col.(7) - Col.(4).

## Discussion

This study examined the differences between community-to-CBO learning and NGO-to-CBO learning strategies for community mobilization among FSW populations. The strength of community mobilization differed significantly over time between the two strategies. Differences are primarily noted with regard to improvements on three parameters: CBO functioning processes, managerial capacities and engagement with stakeholders. The community-to-CBO learning strategy has significantly improved the managerial capacities within CBOs, as compared to the NGO-to-CBO learning strategy. A further examination of which aspects of managerial capacities have improved indicate a significant difference between the two types of learning strategies with regard to the community's capacity to implement, monitor and strategize project services.

An examination of the differences in the CBO functioning process scores by type of strategy shows that the scores related to the existence of CBO leadership and their regular meetings to execute their functions have improved over time under the community-to-CBO strategy. This can be attributed to the direction the project has taken to streamline the institutional systems within the CBO on the community-to-CBO learning strategy. The increased scores on both managerial capacities and CBOs' functioning can be attributed to the review and discussions related to the system gaps in CBO functioning, including but not limited to the executive board and office bearers and their roles within the CBO, and the election process for selecting the leadership team and tackling the unequal power relationships between community organizations and NGOs.

Often spoken about in community mobilization, engagement with the state (such as the police, free public health care, Right To Information [RTI] and other institutions related to social and welfare entitlements) and non-state stakeholders (including goons/rowdies, lodge/shop keepers, drivers, pimps/brothel owners, husbands/partners, worker's unions, women's organizations/NGOs, advocates, media practitioners, politicians, HIV/AIDS care and support institutions, faith-based institutions and neighborhood community members) have also shown improvement under the community-to-CBO learning strategy. The greater understanding on engagement with different stakeholders may have emerged from the experiences of the community faculty from the learning sites. At the program level, beneficiary participants from CBOs also had the opportunity to interact with stakeholders, which helped to build their confidence and motivation. On the other hand, during on-site mentoring, handholding support was also provided in terms of the community themselves conducting stakeholder analysis and designing strategies to engage stakeholders. The role of NGOs in supporting the CBOs in this process cannot be ignored.

It is, however, encouraging that positive changes in many community mobilization parameters are recorded for both types of intervention strategies, and in only one instance, namely, leadership skills, was the NGO-to-CBO strategy more favorable than the community-to-CBO strategy. Although the difference is not statistically significant, one can argue that improvement in leadership skills could be better with experienced agencies guiding CBOs.

While it may be argued that the community-to-CBO learning strategy has shown a positive improvement in building the capacity of CBOs on various parameters, it is important to note that it was primarily the NGOs that built the capacity of community volunteers who were used by the project. Community volunteers, who were mainly champions in the field sites, were drawn from CBOs that had been created and mentored by NGOs. The comparative advantages of learning from the two stakeholders (NGOs and community volunteers) were apparent through the indicators. For instance, for the indicators where objectivity is involved, including accountability, monitoring, leadership selection and reporting, the NGOs have performed relatively better than the community volunteers in transferring skills to CBOs. On the other hand, wherever contextual issues are involved, such as building the capacity of community members on the design and management of interventions, community volunteers have done relatively better than the NGOs in transferring skills to CBOs. This may be because of the ability of community volunteers to understand the processes involved within the CBO structure. The skills that community volunteers have gained from previous experiences in community mobilization have been useful in quicker transferability (within 24 months) as compared to the NGOs. For indicators such as community capacity to organize regular meetings and manage the interventions, NGOs are largely dependent on peer educators from the community whereas, in the case of community volunteers, as they are aware of the community's needs, they can adapt strategies and transfer appropriate skills.

In India, as in other countries, community mobilization has proved valuable for HIV prevention efforts with the female sex worker population [7], [21], [22], [23]. Community-based organizations formed as a result of community mobilization are seen as the natural owners of the intervention and further, can act as trainers to mobilize communities and strengthen other local systems [18], [20]. The current study and its findings provide evidence to the argument from several research findings that new models are needed to demonstrate improved capacity building at the community level [3], [20]. Findings further indicate that on-site capacity building is central to take the participant away from the classroom to the field, thereby providing a real life experience, and shifting the focus of learning from the trainer to the learner. Moreover, it could on occasion even dispense with the trainer and present just the environment and processes, which learners can observe. In summary, the present study shows that the methodology of ‘experiential learning’ is in line with adult learning principles, are suitable for capacity building and could be extended to marginalized communities worldwide.

Although the study findings offer important insights into the comparison between community-to-CBO and NGO-to-CBO learning strategies, the results must be interpreted in light of certain limitations. First, the data drawn from the COPI tool may have missed more complex underpinnings of processes that have community-to-CBO learning strategies, if these were not evident from the indicators. However, these underpinnings are partly explained as possible reasons in the paper, which need validation through further research. Another concern is reporting bias, which we have attempted to minimize by including a validated tool of community mobilization assessment – the COPI – and using independent researchers for the measurement of community mobilization strength. Further, the small sample size used for evaluation of community-to-CBO strategy by using 8 CBOs in the intervention strategy versus only 3 CBOs in the NGO-led strategy, the registration of CBOs at different time points offers a potential bias and limits the generalizability of the findings. While, the bias to different time points of registration of CBOs was mitigated due to the fact that none of the CBOs received any technical support on strengthening of CBOs from NGOs until SAKSHAM project started scaling-up organizational development interventions in 2010. However, larger scale and longer term research studies are needed to confirm the current findings.

In summary, community-led capacity building for strengthening of CBOs is a promising intervention that can be used to scale-up and meet the enormous demand for community mobilization among high risk population groups in India and elsewhere. With the exception of a few measures, many indicators have shown a moderate to high level of improvement if the CBOs were mentored by community volunteers. This does not discount the engagement of NGOs to build the capacity of CBOs, and this paper only argues that the opportunity to use communities as trainers to institutionalize community mobilization is on par with the traditional practice/approach of NGO-led efforts of community mobilization.
